# Genetic Diagnostic Yield and Novel Causal Genes of Congenital Heart Disease

**DOI:** 10.3389/fgene.2022.941364

**Published:** 2022-07-13

**Authors:** Meihua Tan, Xinrui Wang, Hongjie Liu, Xiaoyan Peng, You Yang, Haifei Yu, Liangpu Xu, Jia Li, Hua Cao

**Affiliations:** ^1^ College of Life Sciences, University of Chinese Academy of Sciences, Beijing, China; ^2^ BGI Genomics Co., Ltd, Shenzhen, China; ^3^ NHC Key Laboratory of Technical Evaluation of Fertility Regulation for Non-human Primate, Fujian Maternity and Child Health Hospital, Fuzhou, China; ^4^ College of Clinical Medicine for Obstetrics & Gynecology and Pediatrics, Fujian Medical University, Fuzhou, China; ^5^ Medical Genetic Diagnosis and Therapy Center, Fujian Maternity and Child Health Hospital, Fuzhou, China; ^6^ Fujian Key Laboratory for Prenatal Diagnosis and Birth Defect, Affiliated Hospital of Fujian Medical University, Fujian Maternity and Child Health Hospital, Fuzhou, China; ^7^ Hebei Industrial Technology Research Institute of Genomics in Maternal and Child Health, Shijiazhuang BGI Genomics Co., Ltd, Shijiazhuang, China

**Keywords:** congenital heart disease, genetic etiology, whole-genome sequencing, whole-exome sequencing, diagnostic yield, CHD-related genes

## Abstract

Congenital heart disease (CHD) is the most common congenital malformation in fetuses and neonates, which also represents a leading cause of mortality. Although significant progress has been made by emerging advanced technologies in genetic etiology diagnosis, the causative genetic mechanisms behind CHD remain poorly understood and more than half of CHD patients lack a genetic diagnosis. Unlike carefully designed large case-control cohorts by multicenter trials, we designed a reliable strategy to analyze case-only cohorts to utilize clinical samples sufficiently. Combined low-coverage whole-genome sequencing (WGS) and whole-exome sequencing (WES) were simultaneously conducted in a patient-only cohort for identifying genetic etiologies and exploring candidate, or potential causative CHD-related genes. A total of 121 sporadic CHD patients were recruited and 34.71% (95% CI, 26.80 to 43.56) was diagnosed with genetic etiologies by low-coverage WGS and WES. Chromosomal abnormalities and damaging variants of CHD-related genes could explain 24.79% (95% CI, 17.92 to 33.22) and 18.18% (95% CI, 12.26 to 26.06) of CHD patients, separately, and 8.26% (95% CI, 4.39 to 14.70) of them have simultaneously detected two types of variants. Deletion of chromosome 22q11.2 and pathogenic variants of the *COL3A1* gene were the most common recurrent variants of chromosomal abnormalities and gene variants, respectively. By in-depth manual interpretation, we identified eight candidate CHD-causing genes. Based on rare disease-causing variants prediction and interaction analysis with definitive CHD association genes, we proposed 86 genes as potential CHD-related genes. Gene Ontology (GO) enrichment analysis of the 86 genes revealed regulation-related processes were significantly enriched and processes response to regulation of muscle adaptation might be one of the underlying molecular mechanisms of CHD. Our findings and results provide new insights into research strategies and underlying mechanisms of CHD.

## Introduction

Congenital heart disease (CHD) is a malformation involving structural and functional defects caused by abnormal development of the heart or thoracic and large blood vessels during embryonic development. It is the most common type of congenital malformation and accounts for about 28% of all various kinds of congenital malformations (Van Der Linde et al., 2011). The incidence of CHD in full-term live births is about 8–10/1,000 (0.8–1%), and the incidence of preterm infants may be 10 times higher (about 8.5%) (Jorgensen et al., 2014; Wang et al., 2018). CHD is a complex and highly heterogeneous disease. Its causative factors include environmental factors and genetic or epigenetic factors, but genetic etiologies are considered to be the most important factor. A review study published by Nicholas S. Diab et al. (2021) estimated that genetic etiologies contribute to 90% of CHD cases, although about 55% of them remain unsolved in genetic diagnosis. Chromosomal aneuploidies, copy number variations (CNVs), and damaging variants of disease-causing genes are the mainly diagnosable genetic etiologies (Williams et al., 2019; Buijtendijk et al., 2020).

Chromosomal aneuploidy is the earliest confirmed cause of CHD and accounts for about 9–18% of CHD, and 28–45% of CHD cases diagnosed by a prenatal diagnosis have aneuploidy abnormalities (Mademont-Soler et al., 2013). The application of next-generation sequencing has extended the molecular insights into sub-chromosomal abnormalities, and CNVs have received increased attention as a causative factor of CHD. Deletion at 22q11.2 in DiGeorge syndrome is one of the most common chromosomal abnormalities associated with CHD and is detected in approximately 2% of all CHD patients (Agergaard et al., 2012; Goldmuntz, 2020). Other recurrently affected regions like 15.q11.2, 1.q21.1, and 7q11.23 have also been detected in CHD (Soemedi et al., 2012; Edwards and Gelb, 2016; Mustafa et al., 2020). Rare genetic deletions contribute to about 4% of the population-attributable risk of sporadic CHD (Soemedi et al., 2012) and likely causal CNVs are presumed in 5.6% of CHD patients (Hartman et al., 2011). Cardiac development is a sophisticated process involving a complex regulatory network. Gene encoding transcription factors, cell signal transduction factors, and chromatin modifiers can all interfere with important cell differentiation patterns in heart development, thereby variants disrupting the structure and function of the heart would cause CHD to occur (Lage et al., 2012; Li et al., 2015). Studies have reported that CHD caused by monogenic damaging variants accounts for about 8–11% of CHD cases (Jin et al., 2017; Zaidi and Brueckner, 2017; Diab et al., 2021). At present, a number of CHD pathogenic genes have been located based on family studies, *NKX2.5* (Schott et al., 1998), *TBX5* (Zhang et al., 2020), and *GATA4* (Li et al., 2018) are the most important CHD pathogenic genes. Moreover, whole-exome sequencing (WES) has been routinely used in cohort studies and predicted about 10% of cases are attributable to *de novo* mutations (DNMs) in >400 target genes, including dramatic enrichment for damaging mutations in genes encoding chromatin modifiers (Zaidi et al., 2013; Homsy et al., 2015).

Genetic etiology diagnosis in CHD patients plays an important role in prenatal diagnosis and also in clinical intervention programs. The discovery of novel disease-causing genes or potential association genes and genetic patterns of CHD cohorts is important for the underlying molecular mechanisms of CHD. Unlike previous carefully designed large case-control cohorts by multicenter trials, we designed a reliable strategy in this study to analyze a case-only cohort that could utilize clinical samples sufficiently. This study recruited 121 patients with clinically diagnosed CHD and simultaneously performed low-coverage whole-genome sequencing (WGS) and whole-exome sequencing (WES). The genetic data were manually interpreted and thoroughly bioinformatically analyzed to determine genetic diagnostic yield and potential causative gene prediction. Our findings and results would provide new insights into research strategies and the underlying mechanisms of CHD.

## Materials and Methods

### Patient’s Enrollment and Ethical Conduct of Research

One hundred and twenty-one diagnosed sporadic CHD fetuses or neonates were recruited in Fujian Provincial Maternal and Child Health Hospital from August 2018 to December 2020. The guardians of all involved patients have signed a written informed consent before participating in this project. This study has been approved by the Ethics Committee of the Fujian Maternity and Child Health Hospital (protocol code 2019-147).

### Research Strategy and CHD Relative Genes

Low-coverage WGS and WES were combined to explore the genetic etiologies of CHD patients. The low-coverage WGS was designed to identify chromosomal abnormalities including aneuploidies and CNVs, meanwhile, the WES was utilized to detect the single-nucleotide variants (SNVs) and short insertions or deletions variants (InDels). Considering the high genetic heterogeneity of CHD, we deciphered the genetic causing of CHD not only among the previously reported CHD-related genes but also among other possibly candidate CHD genes following ACMG guidelines. For the CHD-related genes, we manually curated a set of 452 unique genes based on two authoritative reviews of recently published research (Williams et al., 2019; Morton et al., 2022), two large cohort genome-wide association studies (Sifrim et al., 2016; Jin et al., 2017), and 29 case reports retrieved in PubMed (Supplementary Table S1). The retrieved rules in PubMed were: “{[congenital heart ( title)] and [(candidate) or (related) or (relative) or (associated) or (association)]} and [(gene) or (variant)]” and results were filtered by “Case Report” and “Humans”. There were 64 reports preserved, and then the retained studies were subsequently excluded from chromosome abnormalities manually. Finally, 30 CHD genes from 29 case reports were curated.

### Sample Collection and Genomic DNA Extraction

According to the sample condition, cord blood, amniotic fluid, or heart tissues were collected for genomic DNA extraction. Two milliliters of blood were collected in an EDTA tube for cord blood samples, and 20 ml of amniotic fluid was collected and divided into two 15 ml Corning sterilized centrifuge tubes for amniotic fluid samples. Generally, the heart tissue samples were derived from the heart organs of aborted fetuses or muscle tissue from cardiac surgeries. As for the aborted fetuses, we first separated the heart organ and immediately flushed the blood with PBS buffer at 4°C, and then the blood vessels and fat around the heart were cleaned in a sterile environment. Finally, we separated the left and right atrial and ventricle and took more than 100 mg of tissue from each part of the separated tissues. The tissue samples divided from aborted fetuses or cardiac surgeries were all placed in EP tubes and quickly frozen by liquid nitrogen, and subsequently transferred to a −80°C refrigerator for preservation. The genomic DNA (gDNA) was isolated from cord blood or heart tissue utilizing the HiPure Tissue Blood DNA Mini Kit (Magen) and the magnetic bead method amniotic fluid genome extraction kit (NanoMagBio) for amniotic fluid samples. The concentration of gDNA was quantitated using Qubit 2.0 (ThermoFisher Scientific), and the integrity was assessed by agarose gel electrophoresis. The total mass of gDNA was needed more than 1 mg and the integrity of gDNA was required not to be completely degraded.

### Low Coverage Whole Genome Sequencing, Analysis, and Interpretation

The sequencing libraries were performed using MGIEasy Universal DNA Library Prep Set (MGITech) following the standard operating procedures with a total of 50 ng qualified gDNA input. Constructed libraries with sample index were circularized by splint oligo and based on the rolling circle amplification (RCA) mechanism to generate DNA nanoballs for sequencing. More than 0.1X whole-genome coverage raw reads in 35bp plus 10bp (index) were generated for each sample on the MGISEQ-2000 sequencer platform (MGITech). The alignment was conducted by SOAP2 (-v 2 -r 1 -s 25) and the CNVs were detected based on PSCC (population-scale CNV calling) algorithm with default parameters (Li et al., 2014). High-credible CNVs with *p*-values all significant in multiplex statistics test were retained for database annotation and pathogenicity prediction using in-house unpublished software. The annotated databases include disease databases (Decipher, ClinVar), health control individuals database (DGV), and gene-related diseases or syndromes in the structural variant regions in Human Gene Mutation Database (HGMD), and Online Mendelian Inheritance in Man (OMIM). The principle of the unpublished software for pathogenicity prediction was implemented following the ACMG guidelines (Riggs et al., 2020). Comprehensive pathogenicity interpretations were conducted on each variant based on all the previously annotated information. Uncertain significant CNVs with deletion or duplication chromosomal regions containing curated 452 CHD genes were proposed as candidate genetic etiologies of CHD patients, while likely pathogenic and pathogenic CNVs were considered as definitive genetic etiologies of CHD patients.

### Whole Exome Sequencing, Analysis, and Interpretation

A total of 400 ng qualified gDNA for each sample was utilized to conduct sequencing libraries according to the manufacturer’s protocol of MGIEasy Universal DNA Library Prep Set (MGITech), and then the constructed libraries which have sample indexes to be identified were captured using KAPA HyperExome target enrichment kit (ROCHE) or MGIEasy Exome Capture V4 reagent (MGITech) according to their product operating instructions separately. Subsequently, the hybridization capture products were subjected to a post-capture amplification and then circular DNA was generated by the splint oligo ligation. Finally, the DNA nanoball libraries were generated by rolling circle amplification of circularized DNA libraries and were sequenced on the MGISEQ-2000 sequencer platform (MGITech) with a strategy of paired-end 100bp plus 10bp (index). The amount of raw sequencing reads was designed to achieve 100X unique coverage of the exome region. The data pre-processing processes were conducted as previously described (Cao et al., 2020; Belova et al., 2022). Briefly, first low-quality sequencing raw reads were filtered by SOAPnuke v1.5.6 (-Q 2 -G), and then high-quality reads were mapped to human reference genome GRCh37 (hg19) with the BWA-mem algorithm of BWA v0.7.17 (-R “@RG\tID:SampleID\tSM:SampleName\tPL: COMPLETE” -t 2). The alignment SAM files were converted into BAM files and sorted using SAMtools v1.9 (-@ 6 -m 768M). Sorted BAM files were then marked PCR duplications by Picard MarkDuplicates v1.98 (VALIDATION_STRINGENCY = SILENT). Base quality score recalibration was subsequently conducted to correct systematic errors in the base quality scores utilizing the BaseRecalibrator module (--known-sites 1000G_phase1.indels.hg19.sites.vcf --known-sites 1000G_phase1.snps.high_confidence.hg19.sites.vcf --known-sites 1000G_omni2.5.hg19.sites.vcf --known-sites dbSNP_b151_GRCh37p13.vcf --known-sites hapmap_3.3.hg19.sites.vcf --known-sites Mills_and_1000G_gold_standard.indels.hg19.sites.vcf) and ApplyBQSR module (--static-quantized-quals 10 --static-quantized-quals 20 --static-quantized-quals 30 --static-quantized-quals 40 --emit-original-quals) of GATK v4.1.4.0. Short germline variants were detected by the HaplotypeCaller (GATK v4.1.4.0) module with both “vcf” (--dbsnp dbSNP_b151_GRCh37p13.vcf) and “gvcf” (--dbsnp dbSNP_b151_GRCh37p13.vcf -ERC GVCF) formats, and then variants in “vcf” format were filtered by the VariantFiltration module (--filter-expression “QD < 2.0 || MQ < 40.0 || FS > 60.0 || SOR >3.0 || MQRankSum < -12.5 || ReadPosRankSum < -8.0" for SNVs, and --filter-expression “QD < 2.0 || FS > 200.0 || SOR >10.0 || MQRankSum < -12.5 || ReadPosRankSum < -8.0" for Indels). Only variants marked with “PASS” were retained and annotated by the in-house unpublished software. The primary annotation information includes the minor allele frequencies (MAF), protein functional hazard predictions, nucleotide conservation predictions, ClinVar significance records, HGMD records, OMIM records (https://www.omim.org), and pathogenicity predictions. Databases were used for MAF annotation including dbSNP (version 150), the 1,000 human genome dataset (phase 3), the Exome Aggregation Consortium (ExAC, r0.3.1), and the Genome Aggregation Database (GnomAD, r2.0.1). The functional hazard predictions of variants were evaluated by Sorting Intolerant From Tolerant (SIFT), PolyPhen-2 (Polymorphism Phenotyping v2), MutationTaster, and dbscSNV (dbNSFPv2.9.3). The conservation scores were computed based on phyloP and Genomic Evolutionary Rate Profiling (GERP) methods (dbNSFPv2.9.3). ClinVar significance records showed the pathogenicity interpretations of each variant which were submitted in the ClinVar database (version: 2018-08-06 released) and HGMD records supplied the relevant diseases or published papers for each variant, which were archived in the HGMD database (Professional release 2021.1). The OMIM records contained information about phenotypes and their inheritance patterns on mutated genes (https://www.omim.org). Pathogenicity predictions were conducted by in-house software, which was implemented following the ACMG guidelines (Richards et al., 2015). Likely pathogenic and pathogenic variants with their zygosity were consistent with the inheritance pattern recorded in the OMIM database and were manually interpreted for genetic etiologies of CHD patients. Simultaneously, the causative variants were further filtered by the allelic depth of no less than 5X and heterozygous allelic frequency exceeding or equal to 25%. All definitive or candidate genetic etiologies of CHD patients included but were not limited to the curated 452 CHD genes, and meanwhile, we proposed the genes beyond the curated 452 CHD genes were newly candidate CHD relative genes. All definitive or possibly candidate CHD-causing variants were also manually searched on the VarSome website (https://varsome.com/) for pathogenicity prediction.

### Rare Disease Causal Variants Predict and Analysis of Candidate Disease Genes

KGGSeq software is an efficient and comprehensive piece of software that could successfully narrow down whole exome variants to very small numbers of candidate variants in a proband cohort (Li et al., 2012). In this study, we utilized KGGSeq software to narrow down the disease-causing variants based on a 121 CHD cohort and STRING software to conduct functional protein association networks among the candidate disease genes, which are genes from manually interpreted WES and KGGSeq analysis. The bioinformatic pipelines are briefly summarized as follows. First, variants of all 121 samples in “gvcf” format were merged into a single cohort file by the CombineGVCFs module (-A QualByDepth -A RMSMappingQuality -A MappingQualityRankSumTest -A ReadPosRankSumTest -A FisherStrand -A StrandOddsRatio -A Coverage) of GATK v4.1.4.0. Then the joint genotyping was performed by the GATK4 GenotypeGVCFs tool (-A QualByDepth -A RMSMappingQuality -A MappingQualityRankSumTest -A ReadPosRankSumTest -A FisherStrand -A StrandOddsRatio -A Coverage --allow-old-rms-mapping-quality-annotation-data) and the variant quality score recalibration of SNPs and InDels was conducted by the GATK4 VariantRecalibrator module (SNP: -resource:hapmap, known = false, training = true, truth = true, prior = 15.0 hapmap_3.3.hg19.sites.vcf -resource:omni, known = false, training = true, truth = false, prior = 12.0 1000G_omni2.5.hg19.sites.vcf -resource:1000G,known = false, training = true, truth = false, prior = 10.0 1000G_phase1.snps.high_confidence.hg19.sites.vcf -resource:dbsnp, known = true, training = false, truth = false, prior = 2.0 dbSNP_b151_GRCh37p13.vcf -an DP -an QD -an FS -an SOR -an MQ -an MQRankSum -an ReadPosRankSum -mode SNP; InDel: -resource:mills, known = true, training = true, truth = true, prior = 12.0 Mills_and_1000G_gold_standard.indels.hg19.sites.vcf -resource:1000G,known = false, training = true, truth = false, prior = 10.0 1000G_phase1.indels.hg19.sites.vcf -an DP -an QD -an FS -an SOR -an MQ -an MQRankSum -an ReadPosRankSum -mode INDEL) and ApplyVQSR module (--truth-sensitivity-filter-level 99.0), separately. Subsequently, KGGSeq software was utilized to filter variants based on both genotype and variant level qualities and isolate the most promising disease causal candidate variants. Finally, genes with rare disease causal variants were selected for enrichment analysis by STRING software (version 11.5). Meanwhile, we combined the genes with rare disease causal variants and curated 452 CHD genes for potential CHD-related gene analysis. Genes that have interactions with at least 2 curated CHD genes and where the interaction confidence is no less than 0.9 were retained as potential CHD association genes. Enrichment analysis was also conducted on selected potential CHD association genes.

## Results

### Clinical Characteristics of Cohort

There were 121 cases of sporadic CHD diagnosed on fetal or neonatal echocardiogram. Fetal gender was identified with sexual chromosome data distribution which was based on low coverage whole-genome sequencing analysis. In our sporadic CHD cohort, male patients are much more than female patients (57.02% vs. 42.98%) (Table 1). The sample types included in our cohort were tissue, cord blood, and amniotic fluid, which accounted for 56.20, 34.71, and 9.09% of samples, respectively (Table 1). Ultrasound-based clinical diagnosis records were summarized in Supplementary Table S2, and the primary subtypes of CHD were extracted as core clinical features to judge the subgroup of samples. We defined the single CHD subtype as an isolated CHD subgroup and combined CHD subtypes or syndromic forms of CHD as a non-isolated CHD subgroup. Table 1 shows that 51 samples (42.15%) were in the isolated subgroup and 68 samples (56.20%) were in the non-isolated subgroup. The left two samples (1.65%) didn’t have enough information about the CHD subtypes to be classified into any subgroup.

**TABLE 1:** Primary cohort characteristics (*n* = 121 sporadic CHD).

Variable	Outcome
Male, *n* (%)	69 (57.02%)
Female, *n* (%)	52 (42.98%)
Sample type, *n* (%)	
Amniotic fluid	11 (9.09%)
Cord blood	42 (34.71%)
Tissue	68 (56.20%)
The latest ultrasonic testing time period, *n* (%)	
Fetus	79 (65.29%)
Neonate	42 (34.71%)
Clinical features, *n* (%)	
Isolated	51 (42.15%)
Non-isolated	68 (56.20%)
Lack of feature details	2 (1.65%)

### Low Coverage WGS Identified Chromosomal Aneuploidy in 12 Samples and Causative CNVs in Other 18 Samples

Data quality of low-coverage WGS was recorded in Supplementary Table S3. The minimum, maximum, and average unique high-quality reads were 13.51, 56.95, and 32.08M, separately, and their corresponding fetal or neonatal genome depths were 0.16X, 0.66X, and 0.37X. A total of 204 CNVs larger than 100K were identified in 95 samples, and the remaining 26 samples had no micro-deletion or micro-duplication larger than 100K were found (Supplementary Table S4). Among the 204 CNVs, 31.86% (65/204) of the variants have certain clinical significance, and the remaining 68.14% (139/204) of variants have uncertain clinical significance (VUS) (Table 2). Thirteen pathogenic or likely pathogenic chromosome abnormalities were identified in 25 CHD samples, with six chromosome aneuploidies and seven pathogenic or likely pathogenic copy number variants (pCNVs) (Supplementary Table S5). We also analyzed the relative genes contained in the micro-deletion or micro-duplication regions of all the VUS CNVs, and the VUS CNVs were proposed as candidate causative variants if there exist genes in the curated 452 CHD gene set. Based on this analysis, we identified an additional eight candidate CHD disease-causing CNVs involved in 8 samples and 5 of them could be considered as possible genetic etiologies in another 5 samples beyond the 25 mentioned earlier (Supplementary Table S5). All the 21 definitive or candidate CHD causative CNVs could explain the genetic etiology of 30 CHD samples, accounting for 24.79% [95%CI (17.92%–33.22%)] of all samples (Supplementary Table S5), and among the 30 samples, chromosome aneuploidies, pCNVs, and VUS CNVs involving definitive CHD genes could be dependently interpreted as 10, 12, and 5 samples, separately, and the left 3 samples harbor two types of variants. Moreover, three cases of chimera were found in our cohort, they were Trisomy 16 (the chimeric ratio is 12%), del (7q36.2q36.3) (the chimeric ratio is 13%), and del (18q22.3q23) (the chimeric ratio is 14%). Del (22q11.21) is the most frequent causative CNV and can explain the genetic etiology in six CHD samples which occupied 4.96% [6/121, 95%CI (2.07%–10.62%)] of CHD samples. Notably, these six samples all have “Ventricular Septal Defect” in their clinical phenotypes. Trisomy 18, Trisomy 21, and monosomy X are the second most frequent causative CNVs in our cohort, each of them can explain the genetic etiology in 2.48% [3/121, 95%CI (0.53%–7.35%)] of CHD samples (Table 3). Del (15q11.2) was also a recurring CNV that was identified in two CHD samples, accounting for 1.65% [2/121, 95%CI (0.08%–6.20%)] (Table 3). The remaining 16 CNVs were detected once in CHD samples (Table 3).

**TABLE 2:** Functional effects of SNP or InDels and variant classification of CNVs.

Functional effects of SNP or InDels	Variant number (proportion)	Variant classification of CNVs	Variant number (proportion)
Frameshift	606 (83.70%)	Benign	36 (17.65%)
Nonsense	80 (11.05%)	Likely Benign	4 (1.96%)
Span	7 (0.97%)	Variant of Uncertain Significance	139 (68.14%)
Splice-3	16 (2.21%)	Likely Pathogenic	1 (0.49%)
Splice-5	10 (1.38%)	Pathogenic	24 (11.76%)
Stop-gain	5 (0.69%)	Total	204 (100%)
Total	724 (100%)		

**TABLE 3:** Definitive or candidate causative variants of WES and low coverage WGS.

Gene	Sample N	Recurrence variants (samples N)	Chromosome abnormalities	Sample N
*COL3A1*	4	c.5delT (4), c.7_8insC (3)	del (22q11.21)	6
*MED13L*	3	c.1548_1549delCT (3), c.1551_1552insAG (3)	T18	3
*KDR*	3	NA	T21	3
*ANK3*	3	c.4883_4884insCC(2), c.4892_4893delGG (2)	XO	3
*SMAD6*	2	NsA	del (15q11.2)	2
*NIPBL*	2	c.1319delA (2), c.1325C > G (2)	Others^c^	15^d^
*ATP2C1*	2	c.1296_1299delTCTT (2)		
*APC*	2	c.3730_3733delCAAA (2), c.4125delC (2), c.4127_4128insG (2), c.5104G > T (2)		
Others^a^	17^b^	NA		

a 33 genes: ZEB2 (1), WDR35 (1), TTC37 (1), TRPM4 (1), TCAP (1), TAB2 (1), SYNE2(1), SON(1), SHANK3(1), SETBP1(1), RYR2 (1), RAD21 (1), PKP2(1), PEX12 (1), PEX1 (1), NSD1 (1), NONO(1), NODAL (1), NEK1 (1), MYLK (1), MIB1(1), MEGF8 (1), KMT2D (1), KMT2A (1), FREM2 (1), DSP(1), COL5A2 (1), CCDC114 (1), BMPR2 (1), BICC1(1), BBS9(1), BBS2(1), and ARID1A (1).

b 17 samples: 19S5253078R, 20S2200077, 20B10556839, 19B4152086, 19B4152090, 19B4152105, 19B4152110, 19D5253044, 19S5253038R, 19S5253106, 19S5347586, 19S5347592, 20B10556837 (1), 20B10556859, 20S10556845, 20S10556851, and 20S10556862.

c 16 chromosome abnormalities: T9 (1), T13 (1), T16 (1), del (1q42.13q44) (1), dup (4q12q12) (1), del (4q25q25) (1), del (7q11.23) (1), dup (7p12.3) (1), del (7q36.2q36.3) (1), del (11q22.3) (1), dup (12q21.31) (1), dup (14q21.3) (1), dup (17q25.3) (1), del (18q22.3q23) (1), del (18q21.33q23) (1), and dup (Xq28q28) (1).

d 15 samples: 20B10556837, 19B4152096, 19B4152103, 19B4152105, 19B4152086, 19D5253226, 19S5253093, 19S5253099, 20S10556850, 20S10556862, 19B5347543-1, 19S5253089R, 20S10556845, 20S2200077, and 20S2200078.

### WES Reveals Genetic Etiology in 22 CHD Samples and Identified 8 Candidate CHD-Associated Genes

Data quality of WES was also recorded in Supplementary Table S3. The minimum, maximum, and average depths in the capture region were 100.48X, 273.64X, and 171.04X, respectively, and the mean of 10X genome coverage is 98.93% (ranging from 97.4 to 99.5%). After rigorous variant level filtration, we obtained 724 pathogenic or likely pathogenic (P|LP) variants in 268 genes involving 100 samples. The functional effects of the 724 variants are demonstrated in Table2. Frameshift variants are the predominant subtype and account for 83.70%. The functional influence of all the variants can be concluded as protein-truncating variants or loss-of-function variants. Among the 268 genes, there were 33 genes included in the curated 452 CHD gene set, which contained 94 variants and involved 16 CHD samples (Supplementary Table S6). By manually in-depth interpretation, we identified eight additional candidate CHD-causing genes in eight samples, of which six were beside the 16 samples mentioned earlier (Table 4). The eight candidate CHD-causing genes are clear-related genes to syndromes that have cardiac abnormalities. Ultimately, through WES analysis and in-depth manual interpretation, a total of 102 variants in 41 genes were identified and considered as definitive or possibly candidate genetic etiologies in 22 CHD patients, accounting for 18.18% [95%CI (12.26%–26.06%)] (Supplementary Table S6, except variants noted as suspected evidence of compound heterozygous). All 102 variants were protein-truncating variants, including 82 frameshift variants, 13 nonsense variants, five splicing variants, and two stop-gain variants (Supplementary Table S6). Of the 41 CHD disease-causative genes, eight of them repeat in at least two samples (Table 3). The most common causative gene is *COL3A1*, which can explain the genetic etiology in 3.31% [4/121, 95%CI (1.01%–8.47%)] of CHD samples. The *COL3A1* gene variants c.5delT and c.7_8insC recurred in four and three samples, separately. In sample 20S2200088, variant c.7_8insC of the *COL3A1* gene failed in the filtration of allelic frequency, which required the heterozygous allelic frequency to exceed or equal to 25%. However, its allelic frequency is 23%. Moreover, we also found that samples with pathogenic variants in *COL3A1* also detected pathogenic variants in multiple other CHD-related genes. Gene *MED13L*, *KDR*, and *ANK3* are the second most frequent causative genes in our cohort, each of them can explain the genetic etiology in 2.48% [3/121, 95%CI (0.53%–7.35%)] of CHD samples. Except for gene *KDR*, there are recurrence variants in genes *MED13L* and *ANK3*. Gene *SMAD6*, *NIPBL*, *ATP2C1*, and *APC* all have a recurrence in two CHD samples [1.65%, 95%CI (0.08%–6.20%)], and the other three genes, with the exception of *SMAD6*, all have recurrence variants. The remaining 33 CHD disease-causative genes identified in our cohort have appeared only in a single CHD sample.

**TABLE 4:** Candidate CHD-causing genes by manually in-depth interpretation of WES results.

Gene symbol (OMIM ID)	Gene description	Associated phenotype (OMIM ID)	Inheritance	Genotype level	Variants	Sample ID (Zygosity)	ACMG rules	Pathogenicity
*SYNE2* (*608442)	Encodes a nuclear outer membrane protein that binds cytoplasmic F-actin. This binding tethers the nucleus to the cytoskeleton and aids in the maintenance of the structural integrity of the nucleus	Emery-Dreifuss muscular dystrophy 5 (#612999)	AD	3	c.5961-2A > G	19B4152105 (Het)	PVS1 PM2 PP3	P
*MYLK* (*600922)	A muscle member of the immunoglobulin gene superfamily encodes myosin light chain kinase which is a calcium/calmodulin-dependent enzyme. This kinase phosphorylates myosin regulatory light chains to facilitate myosin interaction with actin filaments to produce contractile activity	Aortic aneurysm, familial thoracic 7 (#613780)	AD	3	c.589-2A > G	19S5253038R (Het)	PVS1 PM2 PP3	P
*PKP2* (*602861)	Encodes a member of the arm-repeat (armadillo) and plakophilin gene families. Plakophilin proteins contain numerous armadillo repeats, localize to cell desmosomes and nuclei, and participate in linking cadherins to intermediate filaments in the cytoskeleton. This gene product may regulate the signaling activity of beta-catenin	Arrhythmogenic right ventricular dysplasia 9 (#609040)	AD	3	c.1170+1G > A	19S5253106 (Het)	PVS1 PM2 PP3 PP5	P
*TRPM4* (*606936)	Encodes a calcium-activated nonselective ion channel that mediates transport of monovalent cations across membranes, thereby depolarizing the membrane. The activity of the encoded protein increases with increasing intracellular calcium concentration, but this channel does not transport calcium	Progressive familial heart block, type IB (#604559)	AD	3	c.3504_3514delCGAACAGCGCC(p.Gln1170Serfs*10)	20B10556837 (Het)	PVS1 PM2	LP
*MIB1* (*608677)	Encodes a protein containing multiple ankyrin repeats and RING finger domains that functions as an E3 ubiquitin ligase. The encoded protein positively regulates Notch signaling by ubiquitinating the Notch receptors, thereby facilitating their endocytosis. This protein may also promote the ubiquitination and degradation of death-associated protein kinase 1 (DAPK1)	Left ventricular noncompaction 7 (#615092)	AD	3	c.813G > A (p.Trp271*)	20B10556859 (Het)	PVS1 PM2 PP3	P
*TCAP* (*604488)	Encodes a protein found in striated and cardiac muscle that binds to the titin Z1-Z2 domains and is a substrate of titin kinase, interactions thought to be critical to sarcomere assembly	Cardiomyopathy, hypertrophic, 25 (#607487)	AD	3	c.460_472delCGCTCCCTGTCCC(p.Arg154Alafs*30)	20S10556845 (Het)	PVS1 PM2 PP3	P
*SON* (*182465)	Encodes a protein that contains multiple simple repeats. The encoded protein binds RNA and promotes pre-mRNA splicing, particularly of transcripts with poor splice sites. The protein also recognizes a specific DNA sequence found in the human hepatitis B virus (HBV) and represses HBV core promoter activity	ZTTK syndrome (#617140)	AD	3	c.6754A > T (p.Lys2252*)	20S10556862 (Het)	PVS1 PM2 PP3	P
*DSP* (*125647)	Encodes a protein that anchors intermediate filaments to desmosome plaques and forms an obligate component of functional desmosomes	Arrhythmogenic right ventricular dysplasia 8 (#607450)	AD	3	c.3384_3388delGGAAG (p.Glu1129Glnfs*3)	19S5253078R (Het)	PVS1 PM2 PP3	P
		Dilated cardiomyopathy with woolly hair, keratoderma, and tooth agenesis (#615821)	AD	3				

AD, Autosomal dominant; LP, likely pathogenic; P, pathogenic.

Note: Genotype level 3 means the molecular basis for the disorder is known and at least a mutation has been found in the gene.

### Total Diagnostic Yield of Genetic Etiology in Congenital Heart Disease by Combined WES and Low Coverage WGS

**FIGURE 1:**
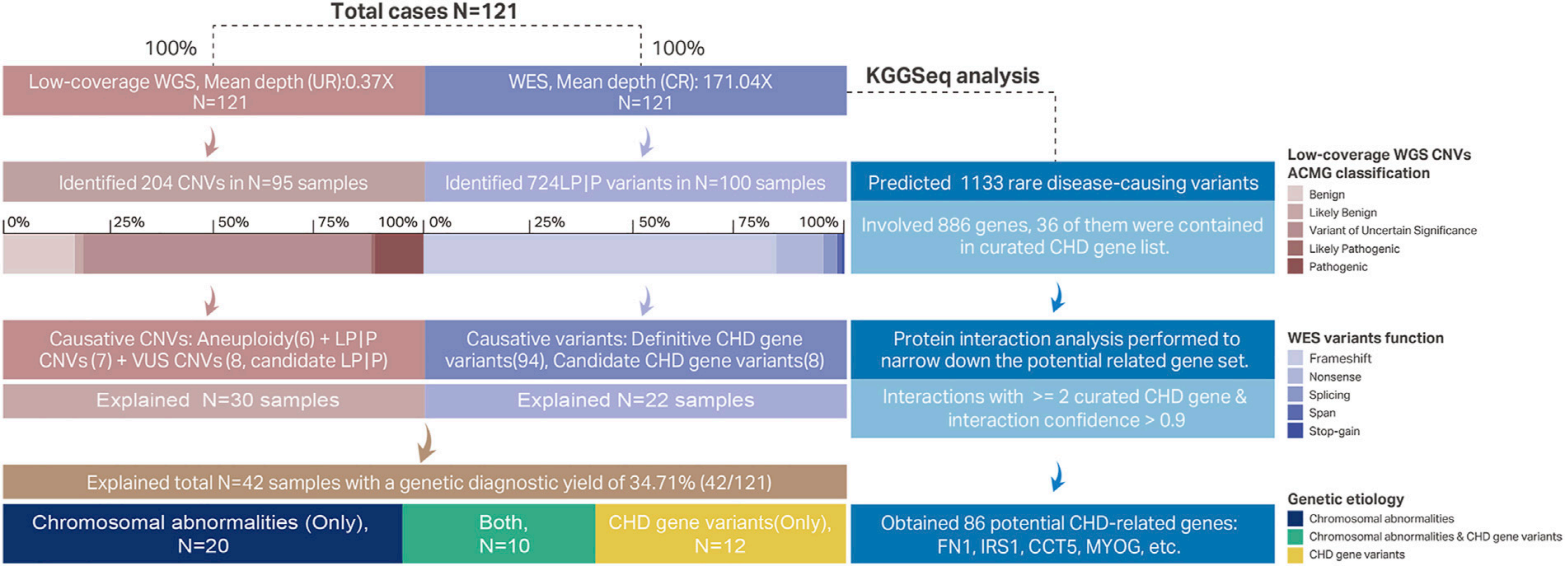
Methodological workflows of genetic etiological diagnosis and CHD-related gene prediction. Each case had conducted low-coverage WGS and WES. The unique reads mean depth of whole-genome by low-coverage WGS was 0.37X. A total of 204 CNVs were identified in 95 samples, and the ACMG classifications of these 204 CNVs were demonstrated in a stacked bar chart. Six aneuploidy variants, seven LP|P CNVs, and eight candidates CHD pathogenic VUS CNVs were proposed as CHD causative CNVs. These 21 causative CNVs all explained 30 samples and some of the causative CNVs were diagnosed repeatedly in multiple samples (Table 3). The mean depth in the capture region of WES was 171.04X. Total 724 P|LP variants were identified in 268 genes involving 100 samples and their functional effect was shown in a stacked bar chart. Among these 724 P|LP variants there were 94 variants belonging to 33 definitive CHD genes and 8 variants belonging to candidate CHD-related genes. Combined with low-coverage WGS and WES, there were 42 samples identified with genetic etiology with a 34.71% diagnostic yield. We also explored the potential CHD association genes by KGGSeq software based on WES data. A total of 1,133 variants on 886 genes were predicted as rare disease-causing. 36 of the 886 were definitive CHD-related genes. The left 850 genes were further narrowed down based on interactions with curated CHD genes. Finally, 86 genes were proposed as CHD-related genes. Abbreviations: UR, unique reads; LP|P, likely pathogenic or pathogenic; CR, capture region.

**FIGURE 2:**
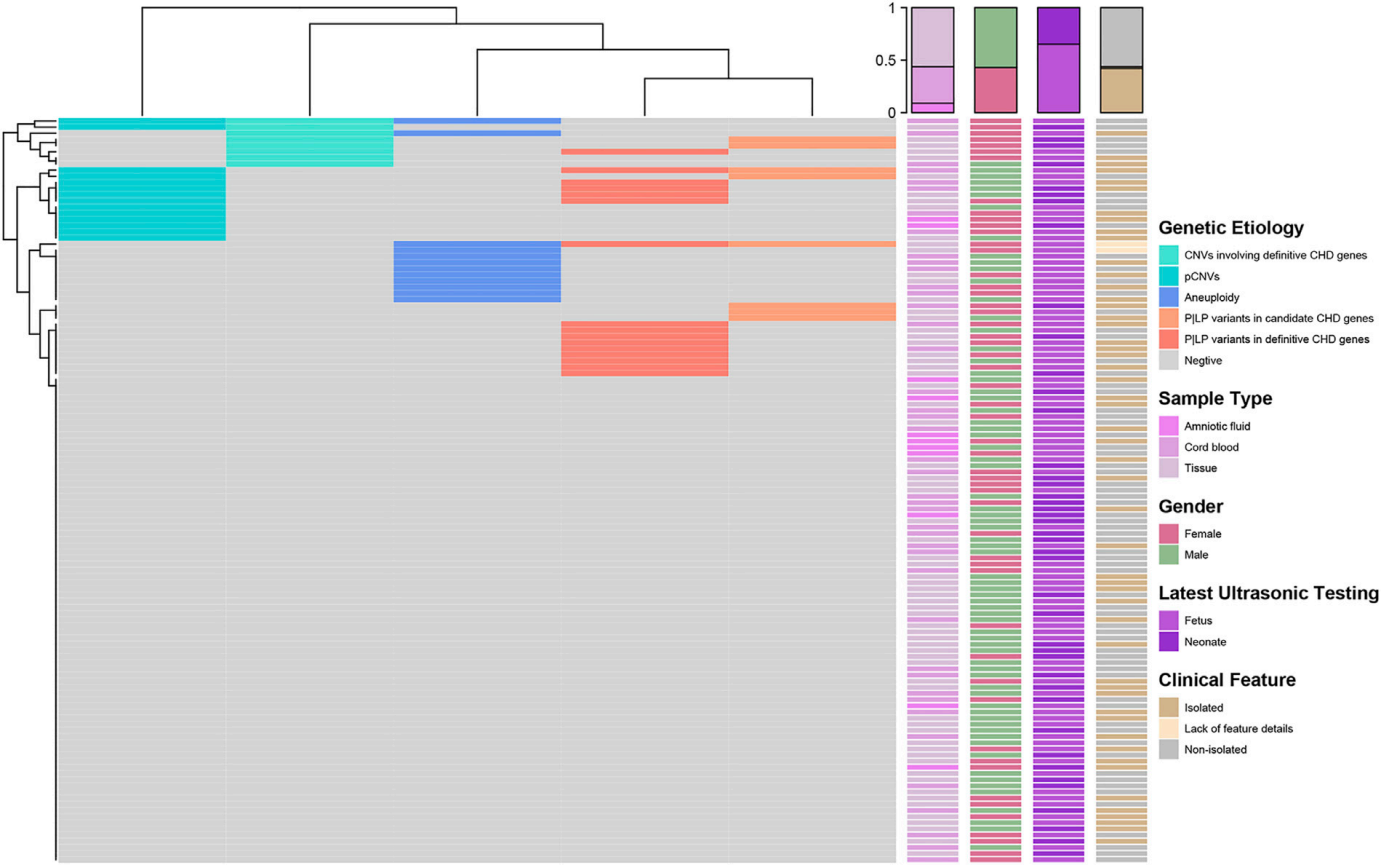
Landscape of distribution of genetic etiology and clinical features (*N* = 121). The heatmap on the left demonstrated the distribution of genetic etiologies detected in each sample. Each row represents a sample and the columns are five categories of genetic etiology. The correspondence colors of genetic etiology were summarized in the right legend. Four clinical characteristics including sample types, gender, latest ultrasonic testing, and clinical features of each sample were demonstrated in the right heatmaps separately. At the same time, we also displayed the distribution statistics of clinical characteristics on the top of the heatmap.

The methodology and processes of genetic diagnosis were summarized in Figure 1. The landscape of the distribution of genetic etiologies and clinical features in our cohort (*N* = 121) was demonstrated in Figure 2. The total diagnostic yield of genetic etiology in our cohort was 34.71% [42/12, 95%CI (26.80%–43.56%)]. Genetic etiologies were divided into five categories based on our analysis strategy: P|LP variants in definitive CHD genes, P|LP variants in candidate CHD genes, aneuploidy, pCNVs, and VUS CNVs involving definitive CHD genes. These five categories of genetic etiology could explain 16, 8, 12, 13, and 8 CHD samples, separately (Supplementary Table S2), and 11 samples have identified two categories of variants, while two samples have identified three categories of variants (Supplementary Table S2). The P|LP variants in definitive or candidate CHD genes were discovered by WES, and the left three categories of chromosome abnormalities were discovered by low coverage WGS. From this perspective, the genetic etiology could be divided into chromosomal abnormalities (low coverage WGS), CHD relative gene variants (WES), and simultaneous harbor chromosomal abnormalities and gene variants, and their diagnostic yield was 16.53% [20/121, 95%CI (10.88%–24.23%)], 9.92% [12/121, 95%CI (5.63%–16.67%)] and 8.26% [10/121, 95%CI (4.39%–14.70%)], separately (Figure 1). The odds ratio (OR) of identifiable genetic etiology was analyzed in subcategories of gender, latest ultrasound periods, and clinical features. Female patients (24/58, 41.38%) have a significantly increased proportion of genetic factors than male patients (18/69, 26.09%) (OR = 2.41, *p*-value = 0.03326). The latest ultrasound testing recordings in the fetus (31/79, 39.24%) have a higher risk of genetic factors than neonates (11/42, 26.19%) but have no statistical significance (OR = 1.81, *p*-value = 0.1662), and isolated CHD patients (21/51, 41.18%) also have a higher but no significant risk of genetic factors than non-isolated CHD patients (19/68, 27.94%) (OR = 1.80, *p*-value = 0.1701).

### Rare Disease-Causing Variants Predicted by KGGSeq and Enrichment Analysis of Potential CHD-Related Genes

**FIGURE 3:**
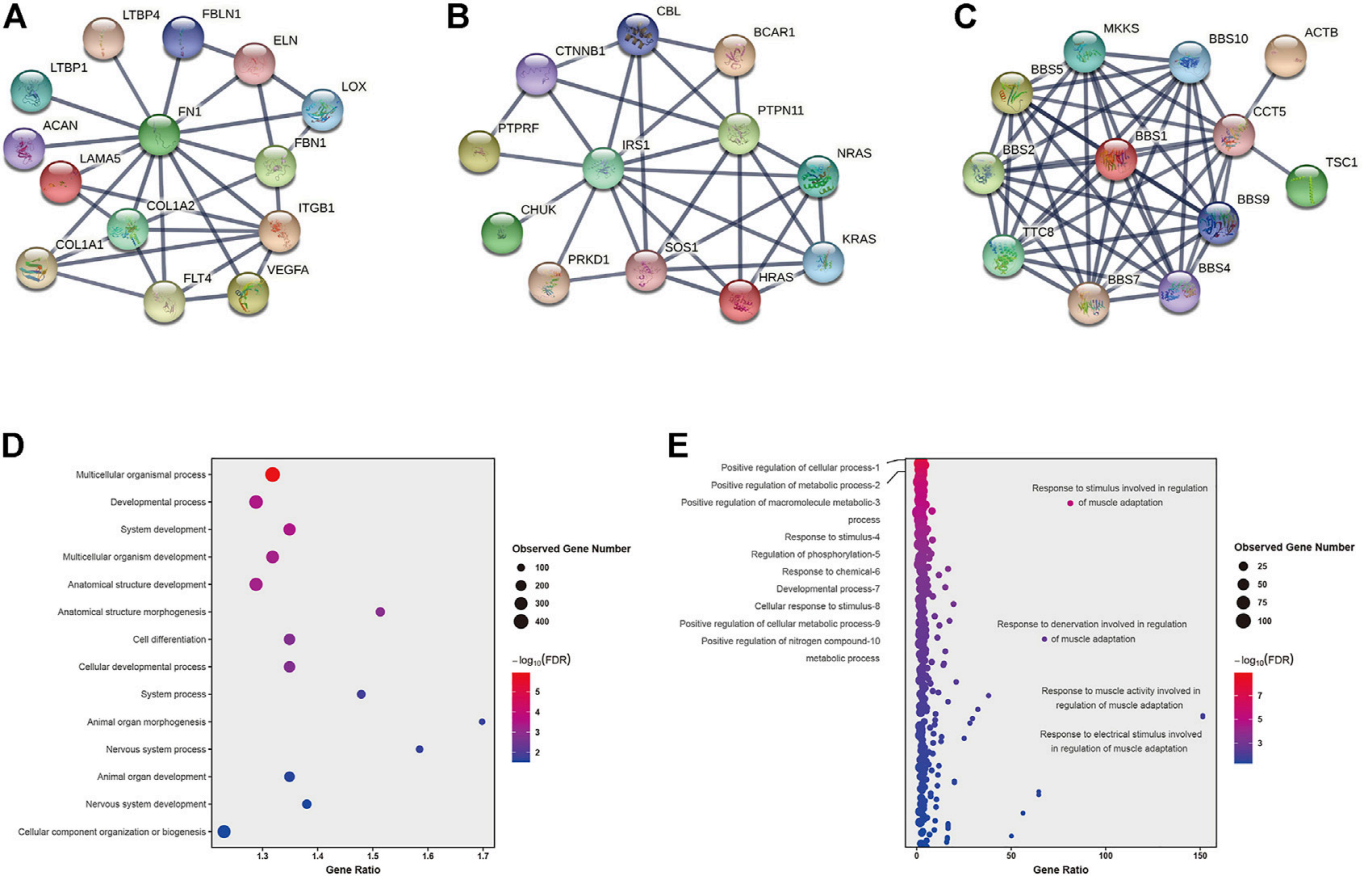
Protein interactions and Genetic causes of CHD identified by WES. **(A–C)**: protein interactions of gene *FN1*, *IRS1*, and *CCT5*. **(D–E)**: Enriched biological processes of 886 genes **(D)** set and 86 genes **(E)** set.

A total of 1,133 variants were predicted as rare disease-causing variants by KGGSeq which involved 886 genes (Supplementary Table S7). Among the predicted 886 genes, 36 of them were contained in the curated 452 genes, and then we combined the remaining 850 genes with the curated 452 CHD genes for protein interaction analysis which aimed to narrow down the targeted potential CHD-related genes. Ultimately, 86 genes that have interactions with at least two curated CHD genes and where the interaction confidence is no less than 0.9 were retained as potential CHD-related genes. Among the 86 potential CHD-related genes, *FN1* has the most associations with definitive CHD genes (13), and genes *IRS1*, and *CCT5* both have the second most interactions with 11 definitive CHD genes (Table S8 and Figures 3A–C). The enrichment analysis of the gene set with 886 genes or 86 potential CHD-related genes was performed using STRING software, and the statistically significant terms with a false discovery rate (FDR) < 0.05 were collected in Supplementary Table S9. Fourteen biological processes by the Gene Ontology resource (Figure 3D) and two KEGG pathways were enriched in the 886 gene set. Multicellular organismal processes (FDR = 1.14E-06), developmental processes (FDR = 0.00036), and system development (FDR = 0.00036) are the top three biological processes, and the two KEGG pathways were ECM-receptor interaction (FDR = 0.0002) and protein digestion and absorption (FDR = 0.021). The 86 potential CHD-related gene set was enriched in 255 biological processes according to the Gene Ontology resource (Figure 3E) and 12 KEGG pathways. The 255 biological processes contained 11 of 14 items of the 886 gene set, which included the top three biological processes mentioned earlier, and the 12 KEGG pathways contained all the two pathways identified in the 886 gene set. The biological processes of 86 potential CHD-related genes were mainly enriched in regulation-related processes, accounting for 51.37% (131/255) of the total significantly enriched biological processes, and the top ten biological processes are listed in Figure 3E. In addition, the bubble plot of biological processes reflected that multiple responses to regulation of muscle adaptation have an extremely high gene ratio (observed number of genes in GO term/expected number of genes in GO term) (Figure 3E). Gene MYOG, a muscle-specific transcription factor that can induce myogenesis in a variety of cell types in tissue culture, is present in all of these processes, as shown in Figure 3E. Among the enriched 12 KEGG pathways, in concordance with the 886 gene set, the ECM-receptor interaction has the most significant FDR (1.80E-10) and three pathways have the equally second most significant FDR (5.14E-05), which include the PI3K-Akt signaling pathway, focal adhesion and dilated cardiomyopathy (Supplementary Table S9). In addition to dilated cardiomyopathy, hypertrophic cardiomyopathy is another significantly enriched cardiac lesion, and its FDR was 0.0024. The gene ratio of dilated cardiomyopathy and hypertrophic cardiomyopathy was 16.60 and 12.88, separately (Supplementary Table S9). In the enrichment results of the molecular functions of 86 potential CHD-related genes, we found the most enriched items were binding functions, which accounted for 68.75% (11/16) of the total significantly enriched items, and the remaining were molecular actively-related functions (3/16) and extracellular matrix structure functions (2/16) (Supplementary Table S9).

## Discussion

In our study, 34.71% [42/121, 95%CI (26.80%–43.56%)] of the samples were identified with a genetic etiology, which is consistent with recently published research such as 34% by Zaidi Samir (Zaidi and Brueckner, 2017) and 34.7% by Mone Fionnuala (Mone et al., 2021). Aneuploidies and CNVs together accounted for 24.79% [30/121, 95%CI (17.92%–33.22%)] of the samples, consistent with published studies around 20%–25% (Zaidi and Brueckner, 2017; Wang et al., 2018; Diab et al., 2021; Morton et al., 2022). Meanwhile, damaging variants of definitive or candidate CHD-associated genes accounted for 18.18% [22/121, 95%CI (12.26%–26.06%)] of the samples, much higher than the previously reported 8%–11% (Jin et al., 2017; Zaidi and Brueckner, 2017; Diab et al., 2021). We speculate this high diagnostic yield was caused by two factors, on one hand, a relatively comprehensive CHD-related gene set was manually curated which explained the genetic etiology for 16 samples (13.22%), on the other hand, the in-depth manual interpretation, we additionally identified eight candidate CHD-associated genes which elevated the diagnostic yield by 4.96% (6/121). Multiple studies consistently estimated that pathogenic variants in at least 400 genes contribute to CHD (Zaidi et al., 2013; Homsy et al., 2015; Jin et al., 2017; Diab et al., 2021), and with the application of next-generation sequencing in large cohort studies, novel CHD-related genes will continue to be identified, which means the elevation of the explainable ratio of CHD.

Aneuploidies were the earliest identified genetic etiology of CHD and most commonly are trisomy 21, 18, 13, and monosomy X (Hartman et al., 2011). Deletion of chromosome 22q11.2 is considered one of the most frequent genetic etiologies of CHD and is detected in approximately 1.9% of all CHD patients (Agergaard et al., 2012). Results of our cohort reflected that deletion 22q11.2 is the most frequent chromosomal abnormality, which explained genetic etiologies in 4.96% (6/121) of all CHD samples. While trisomy 18, 21, monosomy X, and deletion 15q11.2 are both recurrently detected in two samples, which occupied 1.65% of all CHD samples separately. Deletion 15q11.2 was first linked to CHD by Soemedi R. et al. and was found in 0.53% (12/2,256) of CHD samples (Soemedi et al., 2012). In addition to the recurrent chromosomal abnormalities, protein-truncating variants of eight genes were also recurrently identified in our cohort. Gene *COL3A1* (3.31%, 4/121) is the most frequent causative gene, followed by *MED13L*, *KDR*, *ANK3* (2.48%, 3/121), and *SMAD6*, *NIPBL*, *ATP2C1*, and *APC* (1.65%, 2/121). A large cohort GWES study by Sheng C. J. et al. in 2017 showed a totally different recurrently gene pattern that variants in gene *GDF1* account for ∼5% of severe CHD in Ashkenazim, variants in gene *MYH6* accounting for ∼11% of Shone complex, and variants in gene *FLT4* accounting for 2.3% of Tetralogy of Fallot (TOF) (Jin et al., 2017). Another large cohort GWES study by Sifrim et al. (2016) demonstrated that genes *NOTCH1* (0.47%, 4/847), *FBN2* (0.24%, 2/847), *SOS1* (0.24%, 2/847), *NOTCH2* (0.24%, 2/847) recurrently detected protein-truncating variants in non-syndromic CHD cases, while protein-truncating variants in gene *NSD1*, *KMT2A,* and *ADNP* were detected in 0.77% (4/518) syndromic CHD cases separately, and followed by gene *CHD7*, *KMT2D*, and *ANKRD11* in 0.58% (3/518) and gene *MED13L* in 0.39% (2/518) syndromic CHD cases. Multiple studies have shown that different CHD subtypes or cohorts have different patterns of recurrence of chromosomal abnormalities or CHD-associated genes, and by the analysis of the odds ratio of identifiable genetic etiology in gender, latest ultrasound periods, and clinical features, we found female, fetuses, and isolated CHD patients have a higher risk of genetic factors than male, neonates, and non-isolated CHD. The recurrence of monosomy X (in 3 cases) in our cohort might be one of the factors in high OR in female CHD cases. Fetuses diagnosed with CHD included cases not surviving to term in which chromosomal abnormalities were considered major causes (∼60% of spontaneous miscarriage) (Daniely et al., 1998; Li et al., 2020), and the latest follow-up time of isolated CHD cases were focused on the fetal period (78.43%, 40/51). The aforementioned factors and the characteristics of our cohort led to the OR assessment results which reflect the genetic profile of an unselected sporadic CHD cohort.

By in-depth interpretation, we identified and proposed eight candidate CHD association genes: *SYNE2*, *MYLK*, *PKP2*, *TRPM4*, *MIB1*, *TCAP*, *SON*, and *DSP*. They all have defined molecular pathogenic mechanisms associated with the abnormal cardiac phenotype and detected protein-truncating variants that are considered pathogenic or likely pathogenic variants according to ACMG rules. Gene *SYNE2* is linked to Emery-Dreifuss muscular dystrophy (#612999) and its clinical manifestations of proximal muscle weakness, as well as cardiac involvements such as arrhythmias, dilated cardiomyopathy, or heart failure (Heller et al., 2020). Gene *MYLK* is linked to familial thoracic aortic aneurysm (#613780), which manifests clinically as cardiovascular system abnormalities (Wang et al., 2010; Isselbacher et al., 2016). Gene *PKP2* was found to be responsible for arrhythmogenic right ventricular dysplasia 9 (#609040) (Gerull et al., 2004). Gene *TRPM4* is associated with progressive familial heart block, type IB (#604559) (Liu et al., 2010). Gene *MIB1* is the causative gene of left ventricular noncompaction 7 (#615092) (Luxán et al., 2013). Gene *TCAP* is the causative gene of cardiomyopathy, hypertrophic, 25 (#607487) (Hayashi et al., 2004; Bos et al., 2006). Gene *SON* is associated with ZTTK syndrome (#617140), which is a severe multisystem developmental disorder and has congenital defects of the heart in some patients (Kim et al., 2016; Takenouchi et al., 2016). The gene *DSP* causes arrhythmogenic right ventricular dysplasia 8 (#607450) (Yang et al., 2006; Christensen et al., 2010) and dilated cardiomyopathy (#615821) (Norgett et al., 2006; Boulé et al., 2012; Boyden et al., 2016).

In this study, 86 genes were proposed as potential CHD-related genes. The enrichment analysis of these 86 genes displayed that except pathways of cardiac lesions were significantly enriched, including dilated cardiomyopathy and hypertrophic cardiomyopathy, other pathways like ECM-receptor interaction, PI3K-Akt signaling pathway, and Focal adhesion were the top significance of FDR. A research study published by Chen and Jiang (2019) revealed that downregulated differentially expressed genes in arrhythmogenic right ventricular cardiomyopathy (ARVC) of males compared to females were mainly enriched in the “ECM-receptor interaction” and “protein digestion and absorption” pathways. The “protein digestion and absorption” pathway was also enriched in our results with FDR = 0.0316. In a study by Yu et al. (2022) proved that gene *PDTLN1* caused cardiac developmental defects in zebrafish via suppressing the PI3K/AKT signaling pathway, and another study by Zhao et al. (2021) discovered that a high level of melatonin could inhibit growth by inducing apoptosis and cell cycle arrest via PI3K-AKT signaling pathway, thereby interfering with embryonic heart development. In a review by Liu et al. (2019)summarized recent research progresses of actin cytoskeleton in the deployment process of mouse second heart field (SHF) progenitor cells and revealed that actin cytoskeleton played a significant role in mouse SHF development. Regulation of actin cytoskeleton pathway was also enriched in our results with FDR = 0.0022. According to our GO enrichment results, the biological processes of 86 potential CHD-related genes were mainly enriched in regulation-related processes, accounting for 51.37% (131/255) of all significant terms. Generally, the heart is the first functional organ to be developed in vertebrate embryos, and this process is strictly controlled by a gene regulatory network (Saliba et al., 2020). Notably, multiple responses to regulation of muscle adaptation have an extremely high gene ratio in our GO enrichment results, although no relevant studies have been reported so far. Our findings may provide insights for further CHD pathogenic mechanism research.

SNVs in the noncoding regions, particularly those regulating gene expressions, were missed due to insufficient sequencing depth in our study, in which low-coverage WGS was used to detect chromosomal abnormities. A study published by Sweeney et al. (2021) revealed that whole-genome sequencing with about 40-fold diagnosed genetic etiologies in 46% of CHD infants. We can anticipate that a high depth of WGS combined with other omics technologies, such as transcriptome or metabolome, will lead to the discovery of new genetic etiologies and novel insights into the pathogenesis of CHD.

## Data Availability

The data that support the findings of this study have been deposited into CNGB Sequence Archive (CNSA) of China National GeneBank DataBase (CNGBdb) with accession number CNP0003160.
